# Feeding of Infants in Health and Disease

**Published:** 1901-03-02

**Authors:** 


### Hospital Clinics and Medical Progress.

### FEEDING OF INFANTS IN HEALTH AND DISEASE.

Being an abstract of a Paper read by H. Campbell Pope, M.D., at the West London Medico-Chirurgical Society, Friday, February 8th, 1901.

Dr. Campbell Pope said that the subject was one which from its inherent simplicity should be easy to approach and handle, but it was also one which by the evolution of our civilisation, had attained an added complexity, requiring no little patience and much technical knowledge to unravel and elucidate. The problem as presented by nature was simply that of maternal feeding, which required no more care than due respect to the mother's health and the sanitary condition of her person. One of the chief difficulties confronting the practitioner was the wilful shirking of nursing by mothers in a good station and the compulsory abstinence from nursing enforced upon many who had to earn their daily bread. Supposing artificial food were a necessity, three courses were open—the rule of thumb, the guidance of the patent food manufacturer, or a physiological and scientific system. Owing to the efforts of Holt and Rotch, ably seconded by Cautley, Pritchard and others in this country, adequate scientific knowledge on the subject was now available. Rotch set forth two principles: one, that the infant's physiological requirements as to quantity and quality of milk must be met; and secondly, that by proper treatment cow's milk could be so modified as to approximate very nearly to human milk or to any required standard, when by reason of some idiosyncrasy or pathological condition a departure from the normal was indicated. The four important elements of milk were fat, proteid, sugar, and salts. Pritchard says a milk mixture can be prepared by simple means containing these elements in any proportion, and a table had been constructed which contained the percentages which were applicable for infants of all ages and tallied with those of human milk at the corresponding stage of lactation. In America special dairies, known as Walker-Gordon milk laboratories, were largely used, and they had one of them in London. At this laboratory all the modifying of the milk was done, and it was supplied daily to the children of appropriate strength to their age, but by means of Pritchard's table and the Soxhlet steriliser, the same thing could be done at home. If an infant did badly on its food, three points should be noted—the weight, the character of the motions, and the absence or presence of vomiting. In weight, for the first six months, an infant should show a weekly increase of 5-8 ounces. A critical examination of the motions would show which element of the food was disagreeing, and the quantity of such element must be modified in the milk supplied to the infant. The usual cause of vomiting was excess in the quantity of food given at each feeding, and the quantity appropriate to the different ages of infancy was worked out in Pritchard's table. The commonest cause of rickets was overfeeding, and generally in one element of the food, namely, the carbohydrate. Rickety children recovered most rapidly on fresh foods not subjected to boiling. Virol, being a non-cooked food and also anti-scorbutic, from its containing eggshells dissolved in lemon juice, was a valuable addition to the food in such cases. When milk disagreed entirely, a food of meat-juice or egg-albumin in water would be found to meet the difficulty.

*Overfeeding.*—The mother-fed baby ran least risk of overfeeding. In choosing a wet-nurse, overfeeding might be produced by selecting a strong, florid woman to nurse an infant of a pale and delicate mother. Discretion was needed in approximating the foster-mother to the physiological requirements of the nursling. Where the mother's milk was too abundant, it should be drawn off and supplied in measured quantities. The danger of predigested food lay in its easy absorption, and consequent absence of symptoms of overfeeding. When a baby began to perspire much, especially at the back of the neck, it was time to limit its food and see that it obtained more fresh air. In patent foods, the carbohydrate element was generally in excess. Diarrhœa and vomiting were caused by excess of fat and proteid. Only careful watching of the weight and muscular and bony development would indicate when the physiological limit of fat deposition had been reached, showing that the carbohydrate element was in excess.

*Weaning* had sometimes to be accomplished rapidly. In such cases a modified milk diet, adapted to the exact age of the child, should be employed; preferably, weaning was a gradual process. One bottle at night might be given, and at the end of three days, two; and three at the end of six days. Rotch strives to get the infant as rapidly as possible to cow's milk by commencing on modified milk appropriate to the age of the child. Pritchard, on the other hand, says the child should not begin with food appropriate to its age, but on food of a lower standard, whether weaning was intended or not. The nightly bottle should be begun at six months, and weaning should be commenced at the ninth month, even when the child was doing well. Weight should be recorded every week, either by means of Cautley's baby chart or Pritchard's physiological nursery chart.

*Nursery Milk.*—The possible contaminations between udder and mouth were very numerous. Possibly the cow had a sore teat or teats contaminated with fæcal matters. The milkman might have dirty or unhealthy hands and the barn gallons were without dust-protectors—an improvement urgently demanded—and might not be efficiently scalded. Empirical quantities of preservatives were added when the milk reached London; some tablespoonfuls of dirt and dust would be found in each barn gallon; the milk was then served into nursery cans, not always properly sterilised. The danger of non-sterilisation was most real. Dr. Pope had seen both scarlet fever and diphtheria patients drinking out of such cans. The tube of a tube-bottle was almost certainly foul within, and a nurse or mother, possibly with carious teeth, drew the bottle first. Lastly, a pacifier was picked up from the floor and wiped, as likely as not, with a dirty pocket-handkerchief and put into the baby's mouth.

*Feeding of Infants in Sickness.*—Most important was the examination of the motions to see which element of the food was disagreeing and then modifying the milk in accordance. A sick infant, like a sick adult, should be put on a lower scale of diet. Frequently the opposite was done, and the infant, which only began with a slight cold, developed bronchitis from the irritation produced in the bronchial membrane by the excess of metabolic products.

Bronchitis was the commonest cause of death in West London, and he had little doubt that improper feeding, both in young and old, had far more to do with the matter than exposure to cold or any other cause. The infant, when it cried and wished to suck between its meals, was thirsty and wanted water, not food.

*Method and Simplicity.*—It had been perceived in 1883 in France that more method was required in feeding, and the tube-bottle was universally execrated. Notwithstanding that fact, the tube-bottle had yearly massacred more innocents than Herod. An artificially-fed infant must be fed, and not allowed to feed itself. Its food must be apportioned to its physiological needs, not only in quantity and quality, but in the number of feedings per diem and the proper intervals between such feedings. The system of the Walker-Gordon Company by having the milk bottled at the farms in sterilised bottles avoided pollution in transit over long distances, and was the model of the plan which should be adopted in feeding infants. The modification, however, could be done at home, and by using one method for all children simplicity was obtained. Last of all, they must not overlook that maternal nutriment was the best, but in its absence the system advocated was founded on true scientific principles. The first three months of life were of tremendous import to every human being: if the machinery were started wrongly, all vital processes might be vitiated until the end.

